# Development and Preliminary Validation of Chinese Preschoolers’ Eating Behavior Questionnaire

**DOI:** 10.1371/journal.pone.0088255

**Published:** 2014-02-10

**Authors:** Xun Jiang, Xianjun Yang, Yuhai Zhang, Baoxi Wang, Lijun Sun, Lei Shang

**Affiliations:** 1 Department of Pediatrics, Tangdu Hospital, Fourth Military Medical University, Xi’an, Shaanxi, China; 2 Department of Health Statistics, School of Public Health, Fourth Military Medical University, Xi’an, Shaanxi, China; Old Dominion University, United States of America

## Abstract

**Background:**

The objective of this study was to develop a questionnaire for caregivers to assess the eating behavior of Chinese preschoolers.

**Methods:**

To assess children’s eating behaviors, 152 items were derived from a broad review of the literature related to epidemiology surveys and the assessment of children’s eating behaviors. All of these items were reviewed by 50 caregivers of preschoolers and 10 experienced pediatricians. Seventy-seven items were selected for use in a primary questionnaire. After conducting an exploratory factor analysis and a variability analysis on the data from 313 preschoolers used to evaluate this primary questionnaire, we deleted 39 of these 77 items. A Chinese Preschoolers’ Eating Behavior Questionnaire (CPEBQ) was finally established from the remaining 38 items. The structure of this questionnaire was explored by factor analysis, and its reliability, validity and discriminative ability were evaluated with data collected from caregivers of 603 preschoolers.

**Results:**

The CPEBQ consisted of 7 dimensions and 38 items. The 7 dimensions were food fussiness, food responsiveness, eating habit, satiety responsiveness, exogenous eating, emotional eating and initiative eating. The Cronbach’s *α* coefficient for the questionnaire was 0.92, and the test-retest reliability was 0.72. There were significant differences between the scores of normal-weight, overweight and obese preschoolers when it was referred to food fussiness, food responsiveness, eating habits, satiety responsiveness and emotional eating (*p*<0.05). Differences in caregiver’s education levels also had significant effects on scores for food fussiness, eating habits and exogenous eating (*p*<0.05).

**Conclusions:**

The CPEBQ satisfies the conditions of reliability and validity, in accordance with psychometric demands. The questionnaire can be employed to evaluate the characteristics of Chinese preschoolers’ eating behaviors; therefore, it can be used in child health care practice and research.

## Background

World Health Organization (WHO) has reported that the obesity and overweight are important health problems and serious public health challenges in today’s world. The prevalence rate of childhood overweight or obesity has been estimated at 20–40% in European countries [1]. In the United States, results from the 2007–2008 National Health and Nutrition Examination Survey (NHANES) indicated that 10.4% of children aged 2–5 years and 19.6% of children aged 6–11 years were obese, which represented a 25–35% increase since 2000 [2]. In China, the prevalence of obesity and overweight among children aged 0–6 years in 2006 were 7.2% and 19.8%, respectively, with 8.9% and 22.2% for boys, 5.3% and 17.0% for girls. Compared with 1996, the prevalence of obesity and overweight were increased for 3.6 times and 4.7 times respectively [3]. Being obese or overweight in childhood and adolescence has significant effects on the mortalities and morbidities of some diseases later in adulthood. Many metabolic and cardiovascular diseases and some cancers were associated with childhood obesity and overweight [4].

Eating behavior plays a crucial role in the development of childhood obesity and overweight [5]. Schachter *et al* [6] found that responsiveness to satiety was lower in obese individuals and that this decrease in satiety responsiveness might lead to overeating. In some studies, it was reported that over-responsiveness to cues of external food (such as taste, smell and availability), emotions (high enjoyment of food, food responsiveness and emotional overeating), and eating speed were important factors in adiposity development [7], [8]. However, there are conflicting results about the relationship between fussy eating and weight in children in different studies [9]–[11]. For example, eating behaviors are influenced by many factors. Pickiness, anorexia and food refusal were common behaviors among children [12]. According to an epidemiology survey on eating behaviors among children in Beijing [13], Shanghai [14], [15] and Xi’an [16], the prevalence rate of these common problems was above 60%, according to parents’ reports, and ranged from 25% to 40%, according to professionals’ reports.

Lobstein [17] reported that children’s eating behaviors were associated with adolescent and adult’s eating behaviors. The preschool period, when children’s eating behavior problems always occur, might be the crucial time for forming lifelong healthy eating habits [14]–[17]. It has been shown that when children begin to eat independently (approximately 4 years old), their weight was correlated with their eating behaviors [18]. Children begin to learn about eating cultures, adult foods and eating habits, and develop their own eating habits, when they are in preschool [19]. As a result, it is important to identify problems of eating behavior and to cultivate healthy eating habits when a child is in preschool. In this context, an instrument that can help to identify preschoolers’ eating behavior problems and to provide an objective assessment of the severity of these problems is essential for studying eating behavior.

Several standardized tools for assessing children’s eating behaviors have been developed, such as the Dutch Eating Behaviour Questionnaire for Children (DEBQ-C) [20], [21], the Children Eating Behaviour Inventory (CEBI) [22], the Oregon Research Institute Child Eating Behaviour Inventory (ORI-CEBI) [23], and the Children’s Eating Behaviour Questionnaire (CEBQ) [24]. The CEBQ was developed and validated in the UK and has been used in different populations, such as the UK (3–11 years) [25], the Netherlands (6–7 years) [26], Canada (4–5 years) [27], Portugal (3–13 years) [28], Sweden (1–6 years) [29] and China (12–18 months) [30]. All of these instruments are different in structure and have their own merits and applicabilities. For example, the DEBQ-C has 3 dimensions, but the CEBQ has 8 dimensions. The DEBQ-C and the CEBQ can be used to identify and assess the degree of children’s problematic eating behaviors, but the CEBI and the ORI-CEBI are mainly used to identify children’s problematic eating behaviors.

Because of the large differences in race, culture and eating habits among different countries and regions, these instruments are not always effective in all populations. For example, CEBQ has been standardized and adapted into Chinese version [30], but it turned out that the dimensions were not completely accordant with those of the original questionnaire, with ‘food responsiveness’ was split into two factors, ‘Satiety responsiveness’ and ‘Enjoyment of food’ factors were not detected. In recent years, the subject of children’s eating behavior has caught the attention of both experts and parents in China, but most studies have been epidemiology surveys on the prevalence of problematic eating behaviors and their influencing factors. Questionnaires used in these studies are self-constructed and are not tested for reliability and validity, which results in the inconsistence among different studies. To our best knowledge, there is so far no broadly accepted questionnaire for assessing children’s eating behaviors in China. On the above-mentioned basis, the aim of this study is to develop a questionnaire which can be used to evaluate Chinese preschoolers’ problematic eating behaviors.

## Methods

This study was approved by the Research Ethics Committee of the Fourth Military Medical University. All caregivers recruited were provided with written informed consent prior to the collection of any information.

### Literature Review and Primary Questionnaire Development

After reviewing a wide variety of literature related to epidemiologic surveys and the identification of influencing factors of and the evaluation of children’s eating behaviors, published in international or Chinese academic journals in the last 30 years, we established a Chinese item pool including 152 items (Table 1). The item pool was reviewed by 50 caregivers of preschoolers who were selected from the outpatient department of pediatrics of the two largest hospitals in Xi’an and by 10 pediatricians who were familiar with children’s eating behaviors and had performed some research in this field. Each item was critically evaluated, item’s importance (relevance in terms of evaluating eating behavior problems among children) was evaluated by the same ten pediatricians, and item’s frequency (number of cases in which eating behavior problem occurring) [31] was evaluated by the same 50 caregivers. The pediatricians and caregivers scored each item on a Likert-type scale ranging from 1 (not very important/frequent) to 5 (very important/frequent), so each item had 60 scores for important and 60 scores for frequent. Based on the scores assigned by the pediatricians and caregivers, items with a mean score of over 2 were considered to be *very important* and *very frequent*. Items which did not comply with the following percentiles were removed: frequency × importance <50th centile plus importance <50th centile; frequency × importance <50th centile plus frequency <50th centile. The 50th centile was calculated by using the formula of 50th centile = (score_30_+score_31_)/2, score_30_ and score_31_ were the 30th and 31th score of the 60 scores sorted by decreasing order respectively. For example, if the 30th and 31th score (score_30_ and score_31_) for an item’s importance were 2 and 3, it’s 50th centile would be 2.5. After this review, we deleted 75 items that our respondents thought were not common or not important, reducing the item pool to 77 items (Table 2). In order to make all the items unambiguous and readable, two linguistics experts and one psychology expert were then asked to review each item together, and their recommendations were used to develop the primary Chinese Preschooler’s Eating Behavior Questionnaire (CPEBQ).

**Table 1:** Composition and main references of the item pool.

Itemclassification	Definition	Itemnumber	Main references
foodselection	about food selectionbehaviors based on food’s kind, color,smell, taste, textile,shape, etc	19	Wardle J, et al (2001). J Child Psychol Psyc 42(7): 963–70; Archer LA, et al (1991). J Pediatr Psychol 16(5): 629–42; Davies WH, et al (2011). Eating Behaviors 8∶457–63; Seiverling L, et al (2011). Research in Developmental Disabilities 32∶1122–29; Strien TV and Oosterveld P (2008). Int J Eat Disord 41(1): 72–81; Geng G, et al (2009). Appetite 52∶8–14; Dovey TM, et al (2008). Appetite 50∶181–93; Peter M. et al (2005). Int J Eat Disord 38(3): 208–19.; Kristoffer S, et al (2010). Children’s Health Care 39(2): 142–56; Shim JE and Kim J (2011). J Am Diet Assoc 111∶1363–68; Murashima M, et al (2011). Appetite 56∶594–601; Birch LL, et al (2001). Appetite 36(3): 201–10; Jin XM and Shi R (2009). Chin J Child Health Care 17(4): 387–92; Sun J (2008). Med World 9: 66–67; Ren YJ (2011). Health Reading J 4(4): 360–61; Li F and Zhou YC (2010). Chin J Evid Based Pediatr 5(5): 320–33; Zhang XM, et al (2011). Chin J Child Health Care 19(3): 275–77; Jing XM (2010). Chin J Child Health Care 18(7): 537–38.
foodresponsiveness	about the desire to eat foods when he/she see, smell, or supplied, etc.	16	Strien TV and Oosterveld P (2008). Int J Eat Disord 41(1): 72–81; Wardle J, et al (2001). J Child Psychol Psyc 42(7): 963–70; Archer LA, et al (1991). J Pediatr Psychol 16(5): 629–42; Davies WH, et al (2007). Eating Behaviors 8∶457–63; Geng G, et al (2009). Appetite 52∶8–14; Murashima M, et al (2011). Appetite 56∶594–601; Peter M. et al (2005). Int J Eat Disord 38(3): 208–19; Kristoffer S, et al (2010). Children’s Health Care 39(2): 142–56.; Shim JE and Kim J (2011). J Am Diet Assoc 111∶1363–68; Jin XM and Shi R (2009). Chin J Child Health Care 17(4): 387–92; Sun J (2008). Med World 9∶66–67; Ren YJ (2011). Health Reading J 4(4): 360–61; Li F and Zhou YC (2010). Chin J Evid Based Pediatr 5(5): 320–33; Zhang XM, et al (2011). Chin J Child Health Care 19(3): 275–77; Jing XM (2010). Chin J Child Health Care 18(7): 537–38.
eatingspeed	about eating speed ina meal, slowly orquickly, time taking,etc.	12	Wardle J, et al (2001). J Child Psychol Psyc 42(7): 963–70; Archer LA, et al (1991). J Pediatr Psychol 16(5): 629–42; Davies WH, et al (2011). Eating Behaviors 8∶457–63; Seiverling L, et al (2011). Research in Developmental Disabilities 32∶1122–29; Murashima M, et al (2011). Appetite 56∶594–601; Kristoffer S, et al (2010). Children’s Health Care 39(2): 142–56; Jin XM and Shi R (2009). Chin J Child Health Care 17(4): 387–92; Sun J (2008). Med World 9∶66–67; Ren YJ (2011). Health Reading J 4(4): 360–61; Li F and Zhou YC (2010). Chin J Evid Based Pediatr 5(5): 320–33; Zhang XM, et al (2011). Chin J Child Health Care 19(3): 275–77; Jing XM (2010). Chin J Child Health Care 18(7): 537–38.
emotionalfactors ofeating	about eating inresponse to emotions (happy, angry, worrying, depress, etc)	9	Strien TV and Oosterveld P (2008). Int J Eat Disord 41(1): 72–81; Wardle J, et al (2001). J Child Psychol Psyc 42(7): 963–70. Archer LA, et al (1991). J Pediatr Psychol 16(5): 629–42; Davies WH, et al (2007). Eating Behaviors 8∶457–63; Baños RM, et al (2011). Nutr Hosp 26∶890–98; Shapiro JR, et al (2007). Int J Eat Disord 40∶82–89; Jin XM and Shi R (2009). Chin J Child Health Care 17(4): 387–92; Sun J (2008). Med World 9∶66–67; Ren YJ (2011). Health Reading J 4(4): 360–61; Li F and Zhou YC (2010). Chin J Evid Based Pediatr 5(5): 320–33; Zhang XM, et al (2011). Chin J Child Health Care 19(3): 275–77; Jing XM (2010). Chin J Child Health Care 18(7): 537–38.
externalinfluencingfactors ofeating	about the influenceof external factors,such as dishware,food kind, eatingenvironment, otherpeople’s eatingbehaviors, etc.	20	Archer LA, et al (1991). J Pediatr Psychol 16(5): 629–42; Davies WH, et al (2007). Eating Behaviors 8∶457–63; Seiverling L, et al (2011). Research in Developmental Disabilities 32∶1122–29; Baños RM, et al (2011). Nutr Hosp 26∶890–98; Murashima M, et al (2011). Appetite 56∶594–601; Birch LL, et al (2001). Appetite 36(3): 201–10; Jin XM and Shi R (2009). Chin J Child Health Care 17(4): 387–92; Sun J (2008). Med World 9∶66–67; Ren YJ (2011). Health Reading J 4(4): 360–61; Li F and Zhou YC (2010). Chin J Evid Based Pediatr 5(5): 320–33; Zhang XM, et al (2011). Chin J Child Health Care 19(3): 275–77; Jing XM (2010). Chin J Child Health Care 18(7): 537–38.
foodenjoyment	about the enjoymentextent of all kinds offoods	13	Wardle J, et al (2001). J Child Psychol Psyc 42(7): 963–70.; Archer LA, et al (1991). J Pediatr Psychol 16(5): 629–42; Davies WH, et al (2007). Eating Behaviors 8∶457–63; Seiverling L, et al (2011). Research in Developmental Disabilities 32∶1122–29; Davies WH, et al (2007). Eating Behaviors 8∶457–63; Birch LL, et al (2001). Appetite 36(3): 201–10; Jin XM and Shi R (2009). Chin J Child Health Care 17(4): 387–92; Sun J (2008). Med World 9: 66–67; Ren YJ (2011). Health Reading J 4(4): 360–61; Li F and Zhou YC (2010). Chin J Evid Based Pediatr 5(5): 320–33; Zhang XM, et al (2011). Chin J Child Health Care 19(3): 275–77; Jing XM (2010). Chin J Child Health Care 18(7): 537–38.
satietyresponsiveness	about the foodsamount of the childeating in a meal.	14	Strien TV and Oosterveld P (2008). Int J Eat Disord 41(1): 72–81; Wardle J, et al (2001). J Child Psychol Psyc 42(7): 963–70; Archer LA, et al (1991). J Pediatr Psychol. 16(5): 629–42; Davies WH, et al (2007). Eating Behaviors 8∶457–463; Seiverling L, et al (2011). Research in Developmental Disabilities 32∶1122–29; Megumi M, et al (2011). Appetite 56∶594–601; Birch LL, et al (2001). Appetite 36(3): 201–10; Jin XM and Shi R (2009). Chin J Child Health Care 17(4): 387–92; Sun J (2008). Med World 9∶66–67; Ren YJ (2011). Health Reading J 4(4): 360–61; Li F and Zhou YC (2010). Chin J Evid Based Pediatr 5(5): 320–333; Zhang XM, et al (2011). Chin J Child Health Care 19(3): 275–77; Jing XM (2010). Chin J Child Health Care 18(7): 537–38.
desire fordrink	about beveragedrinking	6	Wardle J, et al (2001). J Child Psychol Psyc 42(7): 963–70; Jin XM and Shi R (2009). Chin J Child Health Care 17(4): 387–92; Sun J (2008). Med World 9: 66–67; Ren YJ (2011). Health Reading J 4(4): 360–61; Li F and Zhou YC (2010). Chin J Evid Based Pediatr 5(5): 320–33; Zhang XM, et al (2011). Chin J Child Health Care 19(3): 275–77; Jing XM (2010). Chin J Child Health Care 18(7): 537–38.
snackbehavior	about eatingsnacks aftermeal	11	Archer LA, et al (1991). J Pediatr Psychol 16(5): 629–42; Murashima M, et al (2011). Appetite 56∶594–601; Jin XM and Shi R (2009). Chin J Child Health Care 17(4): 387–92.; Sun J (2008). Med World 9∶66–67; Ren YJ (2011). Health Reading J 4(4): 360–61; Li F and Zhou YC (2010). Chin J Evid Based Pediatr 5(5): 320– 33; Zhang XM, et al (2011). Chin J Child Health Care 19(3): 275–77; Jing XM (2010). Chin J Child Health Care 18(7): 537–38.
activitywhileeating	about activitiesduring the course ofa meal, such aswatching TV, playtoy, etc.	12	Archer LA, et al (1991). J Pediatr Psychol. 16(5): 629–42; Davies WH, et al (2007). Eating Behaviors 8∶457–63; Murashima M, et al (2011). Appetite 56∶594–601; Kristoffer S, et al (2010). Children’s Health Care 39(2): 142–56.; Jin XM and Shi R (2009). Chin J Child Health Care 17(4): 387–92; Sun J (2008). Med World 9∶66–67; Ren YJ (2011). Health Reading J 4(4): 360–61; Li F and Zhou YC (2010). Chin J Evid Based Pediatr 5(5): 320–33; Zhang XM, et al (2011). Chin J Child Health Care 19(3): 275–77; Jing XM (2010). Chin J Child Health Care 18(7): 537–38.
ability ofindependenteating	about the ability ofindependent eating,including take foodsinitially, feeding byhim/herself, etc	7	Archer LA, et al (1991). J Pediatr Psychol. 16(5): 629–42; Seiverling L, et al (2011). Research in Developmental Disabilities 32∶1122–29; Murashima M, et al (2011). Appetite 56∶594–601; Peter M. et al (2005). Int J Eat Disord. 38(3): 208–19; Jin XM and Shi R (2009). Chin J Child Health Care 17(4): 387–92; Sun J (2008). Med World 9: 66–67; Ren YJ (2011). Health Reading J 4(4): 360–61; Li F and Zhou YC (2010). Chin J Evid Based Pediatr 5(5): 320–33; Zhang XM, et al (2011). Chin J Child Health Care 19(3): 275–77; Jing XM (2010). Chin J Child Health Care 18(7): 537–38.
eatinghabits	about behaviorsof chewing,swallowing, spittingout, throwing orhiding foods, etc.	13	Archer LA, et al (1991). J Pediatr Psychol. 16(5): 629–42; Davies WH, et al (2007). Eating Behaviors 8∶457–63; Seiverling L, et al (2011). Research in Developmental Disabilities 32∶1122–29; Murashima M, et al (2011). Appetite 56∶594–601; Peter M. et al (2005). Int J Eat Disord. 38(3): 208–19; Kristoffer S, et al (2010). Children’s Health Care 39(2): 142–56; Jin XM and Shi R (2009). Chin J Child Health Care 17(4): 387–92; Sun J (2008). Med World 9: 66–67; Ren YJ (2011). Health Reading J 4(4): 360–61.; Li F and Zhou YC (2010). Chin J Evid Based Pediatr 5(5): 320–33.; Zhang XM, et al (2011). Chin J Child Health Care 19(3): 275–77.; Jing XM (2010). Chin J Child Health Care 18(7): 537–38.
total		152	

**Table 2:** Items used for “phase 2” and “phase 3”.

1. My child often picks for foods
**2. My child refuses many foods because the food’s taste, smell, appearance, textile, etc.**
**3. My child only eats the foods he/she selected**
4. My child gets full before his/her meal is finished
5. My child chews foods as expected for his/her age
6. My child takes foods between meals without asking
7. Relatives complain my child’s eating
8. My child eats foods that taste different
**9. My child refuses foods he/she did not eat before**
**10. No matter what foods I give him/her during a meal, my child would eat**
**11. My child will throw away or spit out the foods he/she does not like**
**12. My child enjoys all kinds of food**
**13. My child often lose his/her temper because of the meal**
14. It is difficult to satisfy my child’s requirement for meal
**15. Whenever I give him/her foods, my child would eat**
**16. My child wants to eat when he/she smells or see a food**
17. My child finish his/her meal quickly
**18. If permitted, my child would eat continuously**
**19. Although my child is full, he/she could eat more when seeing his/her favorite foods**
**20. My child is always asking for foods**
**21. It seems that the foods I give my child is not enough for him/her every time**
**22. My child can sit before the table and finish his/her meal quickly**
**23. During a meal, my child always leave foods in his/her mouth for a long time and do not swallow**
**24. My child takes more than 30 minutes to finish a meal**
**30. If I do not allow my child play toys, watch TV, or do not tell a story to him/her, he/she would not have his/her meal**
26. If no one playing with him/her, my child would not have his/her meal
**27. My child often eats snacks before a meal, and eats less in the meal**
28. My child takes foods as toy.
29. My child always play while eating
30. If giving a chance, my child would always be having a drink
31. My child always take books or toys to the table when meal
32. My child leaves the table before he/she finish his/her meal
33. My child is interested in tasting foods that he/she has not tasted before
34. My child eats foods and then spit out
35. My child eats more and more slowly during the course of meal
36. My child likes to see outside while eating
37. My child refuses to eat on the table
**38. My child has a good appetite**
**39. Usually, my child would be full after eats a few mouths in a meal**
**40. My child always leave foods in his/her dishes when a meal finished**
**41. My child eats less than other age-matched child**
42. My child often feel hungry before the meal is ready
**43. No matter how much I give him/her, my child would eat completely during a meal**
44. My child wants to eat when he/she see someone eating.
**45. My child would eat more when eating in a restaurant or other’s family**
**46. My child likes compete for foods with others during a meal**
**47. My child would eat more when the foods is changed**
**48. My child would eat more when using his/her favorite dishwares**
**49. My child would be influenced by others when he/she has a meal with other child**
50. My child eats more when happy
**51. My child eats more when angry**
**52. My child eats more when worried**
**53. My child eats more when there is nothing else to do**
**54. My child eats more when he/she makes a mistake**
**55. My child eats more when nobody play with him/her**
56. My child eats more when playing tired
57. My child does not eat when I do not encourage him/her
58. My child will eat when seeing I am not happy
59. My child enjoys all kinds of food
60. My child likes the foods which he/she should not eat
61. My child likes to try new foods
62. My child likes to eat meats
63. My child likes to eat sea-foods
64. My child likes to eat vegetables
65. My child likes to eat fruits
66. My child like to drink beverage
67. My child likes to eat sweet foods, such as candy, ice cream, cake, etc
68. My child likes fast-foods than Chinese foods, such as KFC, etc.
69. My child always wait for meal time
70. My child often refuses to eat during meal time
**71. My child can eat by him/herself**
72. My child swallows foods which being not completely chewed
**73. My child can look for foods by him/herself when he/she feel hungry**
**74. My child needs feeding during a meal**
**75. My child can take foods by him/herself during a meal**
**76. My child can ask for foods initiatively**
77. My child runs around during a meal

Note: (1) Every item of the questionnaire measures the frequency of each problem that has been occurred for the child during the last two weeks. The response format for each item has five levels: 0 = never, 1 = seldom, 2 = sometimes, 3 = often, and 4 = always. (2) All the 77 items were used in phase 2, 38 bold items were used in phase 3.

### Item Response Format and Score Calculation

Every item of the questionnaire measures the frequency of each problem that has occurred for the child during the last two weeks. The response format for each item has five levels: 0 = never, 1 = seldom, 2 = sometimes, 3 = often, and 4 = always.

To calculate eating behavior scores, the mean score was calculated as the sum of the items divided by the number of items answered in each dimension.

### Final Questionnaire Development

Sample 1 consisted of 325 children aged 3–6 years who were randomly selected from one urban kindergarten and one suburban kindergarten in Xi’an with the following inclusion criteria: the child was 3 to 6 years old, his/her caregiver agreed to take part in the survey, and the child suffered from no diseases (e.g., chronic diseases, gastric or intestinal disease, cognitive impairment, etc.) that might influence the child’s appetite or eating behaviors in the past month. Children were excluded from the study if their caregivers were illiterate or reluctant to participate. Caregivers of the selected children were asked to answer the primary CPEBQ under the direction of pre-trained investigators, and 313 complete questionnaires were collected in total. The response rate was 96.3%. This sample was used for item analysis of the primary questionnaire and for constructing the final CPEBQ, which included fewer items.

Variability analysis and principal component analysis (PCA) were performed to reduce the number of items. Parallel analysis [32], [33] was used to identify the number of factors. The entire analysis was performed with SPSS 16.0 software.

### Preliminary Validation of Final Questionnaire

Sample 2 consisted of 653 children aged 3–6 years who were sampled randomly from 3 urban and 2 suburban kindergartens in Xi’an with the same including and excluding criterion as sample 1. Their caregivers were asked to answer the *final questionnaire* under the direction of investigators, and 603 complete questionnaires were collected. The response rate was 92.3%. This sample was used to explore the structure of eating behavior and to evaluate the validity and reliability of the final questionnaire. Ninety caregivers out of the 603 subjects (approximately 15%) who were selected randomly repeated the questionnaire after 2 weeks. There were no differences in gender distribution or age between those who completed and those who did not complete the repeating questionnaire.

Exploratory factor analysis with varimax rotate was performed to extract the factor structure. Confirmatory factor analysis was used to confirm the factor structure of the questionnaire. Reliability coefficients including Cronbach’s *α* coefficient, split-half reliability, and test-retest reliability were calculated to evaluate the reliability of the questionnaire. Pearson’s correlations analysis was used to evaluate content validity and construct validity of the questionnaire. Scores of eating behavior were compared between different groups (gender, age, weight status, and caregiver’s education levels) by using either *t*-tests or one-way ANOVA, A *p* value <0.05 was considered statistically significant. Bonferroni corrections were applied to control multiple testing, A *p* value <0.0083 was considered statistically significant for multiple comparison among age groups, A *p* value <0.0167 was considered statistically significant for multiple comparison among child’s weight status and caregiver’s education levels. The entire analysis was performed with SPSS 16.0 software.

### Data Collection and Quality Control

Caregiver was defined as the primary caregiver who took care of the child’s daily living (diet, sleeping, activity, etc) at home after school and in the weekend. In the investigation, caregiver was asked if he/she was the primary caregiver for the child, if he/she answered yes, this caregiver was selected to complete the survey.

All of the caregivers of the selected children in each kindergarten were asked to congregate in a classroom. Questionnaires were answered by caregivers after an investigator explained the aims and requirements of the study in detail, and the questionnaires were collected by the investigator after the caregivers had finished the questionnaires.

Child’s age, and gender, caregiver’s educational levels, and child-caregiver relationship were also collected from the health record of each selected child; all the health records were stored in the kindergarten. Child’s height and weight were measured at the kindergarten by our investigators according to standardized anthropometric methods [34]. Body mass index (BMI) was calculated using the formula of BMI = weight (kg)/height (m^2^). In accordance with BMI’s reference standard, published by the Centre for Disease Control of China [35], all of the children were classified into three groups: normal weight (age- and sex- specified BMI less than the 85^th^ centile); overweight (age- and sex-specified BMI between the 85^th^ and 95^th^ centiles); and obesity (age- and sex-specified BMI greater than the 95^th^ centile).

All of the surveys were performed by 5 pediatricians who had been engaging in pediatric practice for at least 5 years. All of the investigators were trained prior to administering the questionnaires. All of the questionnaires were double-checked carefully by the primary investigator, and telephone interviews were conducted to fill in missing information when a questionnaire was not answered completely.

EpiData 3.1 software was used to establish a database. To ensure the accuracy of the data, double entry mode was used and a logic check for errors was made.

## Results

### Demographic Characteristics of the Sample

Sample 1 comprised 313 children (161 boys, 152 girls) aged 3 to 6 years (mean age = 4.3±1.4 years). Sample 2 comprised 603 children (322 boys, 281 girls). Within Sample 2, 21.7% of the children were 3 years old, 23.4% were 4 years old, 27.7% were 5 years old and 27.2% were 6 years old. In addition, 10.9% of the children were obese and 12.6% were overweight (Table 3).

**Table 3:** Characteristics of the sample.

		Sample 1 (n = 313)		Sample 2 (n = 603)	
		n	%	n	%
Sex	boys	161	51.4	322	53.4
	girls	152	48.6	281	46.6
Age	3 years	82	26.2	131	21.7
	4 years	76	24.3	141	23.4
	5 years	86	27.5	167	27.7
	6 years	69	22.0	164	27.2
Weight status	normal weight	241	77.0	461	76.5
	overweight	33	10.5	76	12.6
	obesity	39	12.5	66	10.9
Child-caregiver relationship, n (%)	parent	234	74.8	458	76.0
	grandparent	79	25.2	145	24.0
Caregiver’s education	junior high school or less	86	27.5	185	30.7
	senior high school	103	32.9	176	29.2
	college or university	113	36.1	207	34.3
	graduate student	11	3.5	35	5.8

### Item Analysis

The data of Sample 1 were used to decrease the item number. Twenty-five items were deleted because both the standard deviation of their score was lower than 0.85 [36] and their factor loading was lower than 0.4. Fourteen items in which either the score’s standard deviation was lower than 0.85 or the factor loading was lower than 0.4 were deleted because the Cronbach’s *α* coefficient of the questionnaire increased when the item was deleted [36]. Thus, the final CPEBQ consisted of 38 items.

### Structure of the Questionnaire

**Figure 1:**
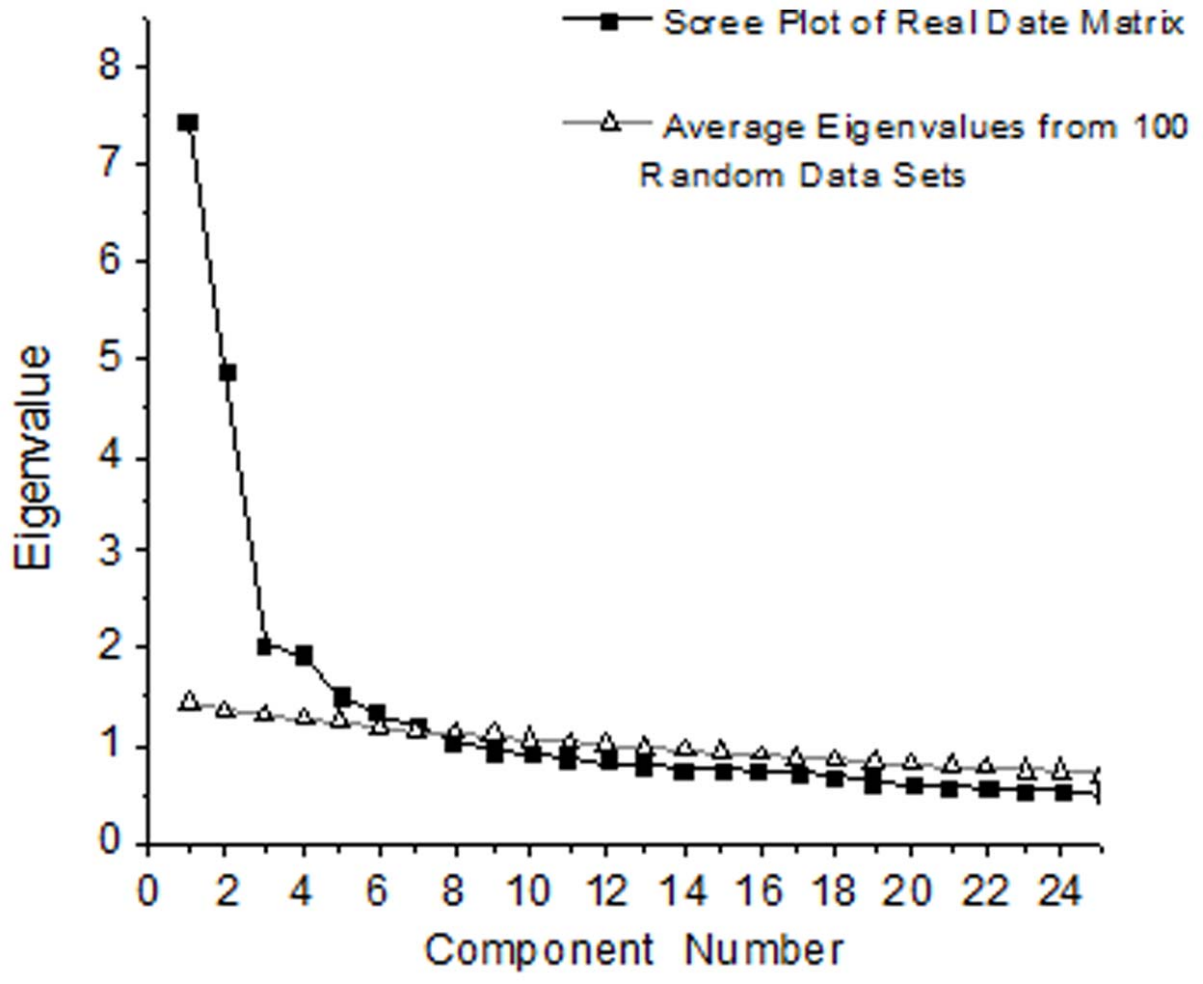
Parallel analysis plot (the first twenty-five factors).

Exploratory factor analysis was performed on 300 complete questionnaires randomly selected from Sample 2. The Kaiser-Meyer-Olkin of sampling adequacy was 0.87, the Approx Chi-Square of Bartlett’s Test of Sphericity was 5633.69, and the probability was lower than 0.05. All of the results indicated that the data were fit for exploratory factor analysis. In the parallel analysis, a random data set was generated on the basis of the same number of items and subjects as the real data matrix. The Scree plot of eigenvalues from the real data was then compared with that from the random data. The point where the two plots cross provides the researcher with a good idea of the absolute maximum number of factors that should be extracted [37]. The parallel analysis plot (Figure 1) showed that 7 factors should be extracted.

The results of the exploratory factor analysis demonstrated that the eigenvalues for these 7 factors were 7.45, 4.88, 2.04, 1.94, 1.51, 1.35 and 1.20, respectively; the variances explained were 19.61%, 12.83%, 5.37%, 5.10%, 3.96%, 3.55% and 3.15%, respectively; and all of the eight factors explained 53.57% of the variance in the 38 items. All of the items’ factor loadings were above 0.4 (Table 4).

**Table 4:** Factor loadings of the factor analysis (Principal Components analysis with Variamax normalized rotation) with all 38 items of CPEBQ.

Dimension name and items	Loading	Dimension name and items	Loading
**Food fussiness, FF(factor 1, 19.61%)**		My child eats less than other age-matched child.	0.74
My child only eat the foods he/she selected	0.75	My child has a good appetite	0.64
My child refuses many foods because the foods’ smell, taste,appearance, texture, etc.	0.72	My child always leave foods in his/her dishes when a meal finished	0.63
My child refuses foods he/she did not eat before	0.69	No matter how much I give him/her, my child would eat completely during a meal	0.52
No matter what foods I give him/her during a meal, my child would eat	0.60	**External eating, EXE(factor 5, 3.96%)**	
My child would throw away or spit out the foods he/she does not like	0.56	My child would eat more when the foods is changed	0.77
My child enjoys all kinds of food	0.48	My child would eat more when using his/her favoritedishwares	0.65
My child often lose his/her temper because of the meal	0.42	My child would eat more when eating in a restaurant orother’s family	0.57
**Food responsiveness, FR(factor2, 12.83%)**		My child likes compete for foods with others during a meal	0.56
Whenever I give him/her foods, my child would eat	0.75	My child would be influenced by others when he/she hasa meal with other child	0.50
Although my child is full, he/she could eat more when seeinghis/her favorite foods	0.74	**Emotional eating, EE(factor 6, 3.55%)**	
If permitted, my child would eat continuously	0.73	My child eats more when angry	0.80
My child is always asking for foods	0.56	My child eats more when he/she makes a mistake	0.73
It seems that the foods I give my child is not enough for him/herevery time	0.52	My child eats more when worried	0.62
My child wants to eat when he/she smells or see a food	0.50	My child eats more when nobody play with him/her	0.59
**Eating habit, EH(factor 3, 5.37%)**		My child eats more when there is nothing else to do	0.52
My child can sit before the table and finish his/her meal quickly.	0.78	**Initiative eating, IE(factor 7, 3.15%)**	
During a meal, my child always leave foods in his/her mouth for along time and do not swallow	0.76	My child needs feeding during a meal	0.73
My child takes more than 30 minutes to finish a meal	0.75	My child can eat by him/herself	0.71
If I do not allow my child play toys, watch TV or do not tell astory to him/her, he/she would not have his/her meal	0.64	My child can take foods by him/herself during a meal	0.54
My child often eats snacks before a meal, and eats less in the meal	0.63	My child can ask for foods initiatively	0.45
**Satiety responsiveness, SR(factor 4, 5.10%)**		My child can look for foods by him/herself when	0.41
Usually, my child would be full after eats a few mouths in a meal	0.75	he/she feel hungry	

The structure of the questionnaire was further evaluated by using confirmatory factor analysis on the data of the remaining 303 subjects of Sample 2. A robust maximum likelihood (ML Robust) estimation procedure, which is robust to violations of normality, was used to test the model. The model was evaluated with five pre-established criteria: (a) a normed fit index (NFI) of 0.90 or higher; (b) a non-normed fit index (NNFI) of 0.80 or higher; (c) a robust comparative fit index (R-CFI) of 0.90 or higher; (d) a root mean square residual (RMSR) of 0.05 or lower; and (e) a Satorra–Bentler scaled statistic (SB-*χ* ^2^/*df*) ratio of 2.0 or lower [38], [39]. The results indicated that 7 dimensions of the questionnaire provided good fit for the data (NFI = 0.88, NNFI = 0.91, R-CFI = 0.92, RMSR = 0.04, and SB-*χ* ^2^/*df* = 1.79).

### Factors Explanation

To understand the potential meaning of the items in each factor, we established the specific definition for each factor. Factor 1 contained 7 items and was named “food fussiness (FF).” It could be applied to assess whether a child refuses food because of its smell, taste, appearance or texture. Factor 2 contained 6 items and was named “food responsiveness (FR).” It could be used to assess children’s desire to eat food. Factor 3 contained 5 items, which reflect unhealthy eating habits such as playing or watching TV while eating or waiting a long time to eat. As a result, we labeled it as “eating habit (EH).” Factor 4 contained 5 items and was named “satiety responsiveness (SR).” It measures satiety sensitivity. Factor 5 contained 5 items and was named “exogenous eating (EXE).” It helps to evaluate responses to external factors that can influence eating. Factor 6 contained 5 items and was named “emotional eating (EE).” It is used to assess the eating status of a child when he or she is experiencing negative emotions. Factor 7 contained 5 items and was named “initiative eating (IE).” It measures the ability of children to eat independently.

### Reliability

All of the 603 subjects of Sample 2 were included in an internal reliability analysis. The Cronbach’s α coefficient for the questionnaire was 0.92, and the 7 dimensions ranged from 0.74 to 0.87. The Guttman split-half reliability of the questionnaire was 0.86, and the 7 dimensions ranged from 0.69 to 0.85. The two-week test-retest reliability for the questionnaire (n = 90) was 0.72, with the 7 dimensions ranging from 0.58 to 0.81 (Table 5).

**Table 5:** Reliability coefficient of CPEBQ.

Dimension	Cronbach’s *a*	Guttman Split-Half reliability	Test-retest reliability
FF	0.87	0.82	0.74
FR	0.77	0.69	0.73
EH	0.81	0.85	0.78
SR	0.74	0.80	0.69
EXE	0.80	0.73	0.79
EE	0.78	0.76	0.58
IE	0.79	0.81	0.81
Total	0.92	0.86	0.72

### Validity

All of the items were based on a deep understanding of a variety of studies related to children’s eating behaviors published in international or Chinese academic journals, and carefully reviewed twice by 10 experienced pediatricians and 50 caregivers. The experts’ consistency coefficients in each round were 0.327 and 0.358, respectively. All of these measures would strengthen the content validity of the questionnaire. The items of the questionnaire were easy to understand, and the mean time for completing a survey was 23±1.7 minutes.

Correlations between the dimensions of the questionnaires were shown in Table 6. The results suggested that the questionnaire clustered in a fairly coherent way. Dimensions of positive eating (food responsiveness, exogenous eating, emotional eating, initiative eating) tended to be positively correlated to and be negatively correlated to dimensions of negative eating (food fussiness, eating habit, satiety responsiveness). The significant correlations between dimensions were moderate. These results also indicated that the questionnaire had good construct validity.

**Table 6:** Pearson’s correlations between dimensions of CPEBQ (n = 603).

Dimension	FF	FR	EH	SR	EXE	EE	IE
FF	1.00	–	–	–	–	–	–
FR	−0.35**	1.00	–	–	–	–	–
EH	0.18	−0.17	1.00	–	–	–	–
SR	0.41**	−0.47**	0.37**	1.00	–	–	–
EXE	−0.16	0.52**	−0.29**	−0.42**	1.00	–	–
EE	−0.33**	0.29**	−0.15	−0.39**	0.28**	1.00	–
IE	−0.23*	0.41**	−0.16	−0.40**	0.32**	0.25*	1.00

Note: Data listed in this table were Pearson correlation coefficient; **p<0.01 *p<0.05.

### Discriminating Ability

Table 7 showed the score by gender, age, weight status and mother’s educational level. Boys and girls did not differ in the score of each dimension (*p*>0.05). Scores for unhealthy eating habits decreased with age (*p*<0.05). Scores for food fussiness, food responsiveness, eating habits, satiety responsiveness and emotional eating showed significant differences among normal-weight, overweight and obese preschoolers (*p*<0.05). The scores of eating habits and exogenous eating showed significant differences among caregiver’s educational levels (*p*<0.05).

**Table 7:** Comparison of eating behavior scores () among different groups (n = 603).

	FF	FR	EH	SR	EXE		EE		IE	
Gender										
Boys	2.28±0.77	2.47±0.47	2.31±0.62	3.01±0.58	2.81±0.79		3.02±0.67		2.79±0.68	
Girls	2.32±0.75	2.51±0.49	2.19±0.63	3.02±0.59	2.87±0.74		2.98±0.65		2.82±0.73	
Age (years)										
3	2.34±0.81	2.52±0.46	2.35±0.64	3.06±0.53	2.76±0.72		2.94±0.63		2.79±0.70	
4	2.36±0.79	2.53±0.47	2.36±0.63	3.04±0.57	2.77±0.74		3.06±0.69		2.81±0.66	
5	2.29±0.78	2.54±0.52	2.12±0.65^a^^,^ b	3.05±0.64	2.77±0.78		2.85±0.67		2.67±0.69	
6	2.38±0.69	2.56±0.50	2.05±0.54^a^^,^ b	3.05±0.62	2.76±0.69		2.95±0.72		2.83±0.68	
Weight status										
NW	2.25±0.70	2.56±0.58	2.04±0.78	3.16±0.57	2.89±0.76		2.87±0.66		3.75±0.79	
OW	2.69±0.69^c^	2.73±0.57	2.97±0.75 c	2.66±0.59^c^	2.95±0.75		2.89±0.72		3.81±0.77	
OB	3.36±0.72^c^^,^ d	3.52±0.61^c^^,^ d	3.22±0.76^c^	2.09±0.62^c^^,^ d	3.02±0.75		3.21±0.65^c^^,^ d		3.68±0.78	
Caregiver’s education level										
JHS	2.29±0.83	2.59±0.51	2.48±0.72	3.07±0.65	2.64±0.72		3.07±0.67		2.74±0.69	
SHS	2.38±0.76	2.46±0.47	2.28±0.65^e^	2.96±0.57	2.77±0.79		3.02±0.65		2.85±0.73	
CU	2.26±0.77	2.50±0.48	2.17±0.69^e^	3.02±0.59	2.94±0.70^e^		2.89±0.63		2.77±0.65	

Note: 1. NW = normal weight, OW = overweight, OB = obesity; JHS = junior high school or less, SHS = senior high school, CU = college or university or graduate student; 2. t-test were performed to compare the difference in the mean scores between boys and girls, and One-way ANOVA were performed to compare the difference in the mean score between different age, weight status and caregiver’s educational level groups. Bonferroni corrections were applied to control multiple testing; 3.

a *P*<0.0083 vs 3 years,

b *P*<0.0083 vs 4 years; 4.

c *P*<0.0167 vs NW,

d *P*<0.0167vs OW; 4.

e *P*<0.0167 vs JHS,

f *P*<0.0167 vs SHS.

## Discussion

In this study, we have developed a Chinese Preschoolers’ Eating Behavior Questionnaire, which consisted of 38 items and 7 dimensions: food fussiness, food responsiveness, eating habit, satiety responsiveness, exogenous eating, emotional eating and initiative eating. This questionnaire is of good reliability and validity, and therefore could be used to study preschoolers’ eating behaviors in China.

Eating behavior is affected by many factors, and children behave differently in regards to these factors. So, it is very difficult to accurately measure eating behavior. Although several countries or regions have developed a variety of standardized tools to identify and assess children’s eating behavior, a commonly recognized structure of questionnaires for evaluating children’s eating behavior has yet to be further established. In addition, the questionnaires often focus on different populations. For instance, the DEBQ-C was developed for Dutch children aged 7–12 years, and it included 20 items and 3 dimensions: emotional eating, restrained eating and exogenous eating [20], [21]. The CEBQ included 8 dimensions: responsiveness to food, enjoyment of food, satiety responsiveness, slowness in eating, fussiness, emotional overeating, emotional under-eating, and desire for drinks [24]. Our questionnaire, in accordance with the DEBQ-C, included dimensions of emotional eating and exogenous eating and also included 5 other dimensions. In comparison with the CEBQ, we could not extract dimensions that reflect a desire for drinks, slowness in eating and enjoyment of food, but we extracted dimensions of eating habits and initiative eating, which are not included in the CEBQ. All of these differences might reflect that children’s eating behaviors are different among different countries, regions, and age groups.

The development of children’s food preferences involves a complex interplay of genetic, familial, and environmental factors [40]. Emerging evidence suggests a strong genetic influence on appetites in children, but environmental factors may also play an important role in shaping children’s eating behaviors. This study focused on the eating behaviors of Chinese children aged 3 to 6 years. Among the 7 dimensions in the present questionnaire, only the score for “eating habits” demonstrated decreasing trends with age, and we did not observe significant differences between the scores of different age groups in other dimensions. These results were consistent with the results of Farrow’s study of the CEBQ of UK children aged 2–5 years [41], which showed that eating behavior has traits of continuity and stability in children. Our results also indicated that boys and girls did not differ in their eating behavior score, which was consistent with the results of the CEBQ study of Swedish children aged 1–6 years [29]. It has been reported that boys and girls have different eating styles among adolescents. However, it is not known at what age these differences start to develop [42]. Nakao’s study [18] showed that when a child (approximately 4 years old) begins to eat independently, his or her weight is correlated with his or her eating behaviors. Our study indicated that the scores of food fussiness, food responsiveness, eating habits, satiety responsiveness and emotional eating revealed significant differences among normal-weight, overweight and obese preschoolers, which was consistent with Nakao’s study [18] and the study of the CEBQ in Canadian children aged 4–5 years [27]. All of these results supported that our questionnaire had good discriminate validity.

The common eating behaviors among children under 6 years old in China reported by studies [13]–[16], [43] were eating less (10.3∼41.3% ), eating slow (27.4∼46.4% ), anorexia (6.6∼30.5%), food refusal (11.0∼22.6%), unwilling to try new foods (17.4∼33.3%), favorite some foods with special type, texture, color, etc (17.4∼48.2%), doing other thing while eating (14.0–60.1%), no fixed eating site (9.1∼45.8%), eating influenced by emotion (8.6∼16.8%), eating influenced by external factors (13.7∼36.9%), etc. CPEBQ developed by our study, which consisted of 7 dimensions: food fussiness, food responsiveness, eating habit, satiety responsiveness, exogenous eating, emotional eating and initiative eating. We thought the 7 dimensions of CPEBQ could cover all the reported common eating behaviors among preschool children in China.

Eating behavior evaluations are critically informative in developing clinical management programs that provide comprehensive intervention and education for children with eating behavior problems and their families. Higher score in any dimension of CPEBQ mean the child is more likely having the eating behavior problem in this aspect. Using CPEBQ as a foundation, pediatricians or child health care workers could develop targeted support services for children and their families, such as medical education about eating behavior disorder and feeding counseling.

Of note, this study has several limitations that should be acknowledged. First, the subjects might not be completely representative of the entire population of Chinese preschool children because they were only selected in Xi’an. Second, we did not test the criterion-related validity of CPEBQ because there were not a general accepted assessment tool for children’s eating behavior in China. We will further test this questionnaire’s reliability, validity and measurement invariance in other populations, and improve it to become a more generic questionnaire for eating behavior assessment among preschoolers in China.

## Conclusions

We have developed a Chinese Preschoolers’ Eating Behavior Questionnaire. To our knowledge, this is the first time that a Preschooler’s Eating Behavior Questionnaire has been developed for assessing the eating behaviors of preschool children in China. It satisfies all psychometric properties. Therefore, this questionnaire could be used as a practicable tool for evaluating preschool children’s eating behavior in China.
